# Minimum redundancy maximal relevance gene selection of apoptosis pathway genes in peripheral blood mononuclear cells of HIV-infected patients with antiretroviral therapy-associated mitochondrial toxicity

**DOI:** 10.1186/s12920-021-01136-1

**Published:** 2021-12-01

**Authors:** Eliezer Bose, Elijah Paintsil, Musie Ghebremichael

**Affiliations:** 1 Massachusetts General Hospital Institute of Health Professions, Boston, MA USA; 2 Department of Pediatrics, Yale University School of Medicine, New Haven, CT USA; 3 Harvard Medical School, Cambridge, MA USA; 4 Ragon Institute of MGH, MIT and Harvard, 400 Technology Square, Cambridge, MA 02129 USA

**Keywords:** HIV, Apoptosis, Antiretroviral therapy, Mitochondrial toxicity, Machine learning, Minimum redundancy maximum relevance (mRMR)

## Abstract

**Background:**

We previously identified differentially expressed genes on the basis of false discovery rate adjusted *P* value using empirical Bayes moderated tests. However, that approach yielded a subset of differentially expressed genes without accounting for redundancy between the selected genes.

**Methods:**

This study is a secondary analysis of a case–control study of the effect of antiretroviral therapy on apoptosis pathway genes comprising of 16 cases (HIV infected with mitochondrial toxicity) and 16 controls (uninfected). We applied the maximum relevance minimum redundancy (mRMR) algorithm on the genes that were differentially expressed between the cases and controls. The mRMR algorithm iteratively selects features (genes) that are maximally relevant for class prediction and minimally redundant. We implemented several machine learning classifiers and tested the prediction accuracy of the two mRMR genes. We next used network analysis to estimate and visualize the association among the differentially expressed genes. We employed Markov Random Field or undirected network models to identify gene networks related to mitochondrial toxicity. The Spinglass model was used to identify clusters of gene communities.

**Results:**

The mRMR algorithm ranked DFFA and TNFRSF1A, two of the upregulated proapoptotic genes, on the top. The overall prediction accuracy was 86%, the two mRMR genes correctly classified 86% of the participants into their respective groups. The estimated network models showed different patterns of gene networks. In the network of the cases, FASLG was the most central gene. However, instead of FASLG, ABL1 and LTBR had the highest centrality in controls.

**Conclusion:**

The mRMR algorithm and network analysis revealed a new correlation of genes associated with mitochondrial toxicity.

## Background

Although current antiretroviral therapy (ART) has reduced HIV-associated morbidity and mortality [1–4], ART-associated toxicity is still pervasive in people living with HIV (PLWH) [5–7]. A recent study from Italy showed that the 1-year probability of discontinuation of ART due to toxicity was 19% for patients who initiated ART between 2008 and 2014 [8]. All classes of antiretroviral drugs are associated with toxicity. Nucleoside reverse transcriptase inhibitors (NRTIs), the first class to show anti-HIV activity, are associated with toxicities such as skeletal muscle myopathy, lactic acidosis, lipodystrophy, peripheral neuropathies, cardiomyopathies, and pancytopenia [9–12]. These toxicities are due to NRTI-induced mitochondrial dysfunction through the inhibition of mitochondrial DNA (mtDNA) polymerase gamma (Pol-γ) [13]. Recently, Pol-γ independent mitochondrial dysfunction has been associated with several components of ART [14–16]. For example, protease inhibitors (PIs) and non-nucleoside reverse transcriptase inhibitors (NNRTIs) do not inhibit Pol-γ, and yet they cause toxicities commensurate with mitochondrial dysfunction [17, 18]. Although the underlying mechanisms are not well understood, most ART classes can cause apoptosis, a mitochondrion function [19, 20]. Thus, apoptosis biomarkers could be used potentially to diagnose and monitor ART-associated toxicity. We recently reported that in a case–control study (HIV + with mitochondrial toxicity vs. HIV uninfected controls), a total of 26 of 84 genes of the apoptosis pathway were differentially expressed [21].

The objectives of the current study, a secondary data analysis, were twofold: First, we sought to select the most relevant and least redundant genes in the differential expression profile of the apoptosis pathway in HIV-infected patients with ART-associated mitochondrial toxicity (cases) versus HIV-uninfected individuals (controls). We employed the maximum relevance minimum redundancy algorithm on the 26 differentially expressed genes between the cases and controls. This algorithm performs better than the differential gene expression analyses we had previously conducted, for the latter failed to account for redundancy between the selected genes. Several classification algorithms, including Linear Discriminant analysis, Quadratic Discriminant analysis, k-nearest neighbor, Support Vector Machine, Classification trees, Adaboost, Neural Networks, Random forest, Gaussian process, and Logistic Regression were used to assess the prediction accuracy of the mRMR genes. Second, we conducted network analyses to estimate and visualize complex associations among the genes differentially expressed between cases and controls. More specifically, we employed Markov Random Field (MRF) or undirected network models to identify network structures related to mitochondrial toxicity. That is, to examine how HIV alters the protective network structure of genes in the control group (perturbations to the protective network structure of genes in controls). The Spinglass model was used to identify clusters of gene communities in cases and controls. Permutation based test was used to compare the networks of cases and controls.

## Methods

### Study design and participants

This study is a secondary analysis of data obtained from a previous case–control study comprising of HIV-infected individuals with mitochondrial toxicity (cases, n = 16) and HIV uninfected individuals (controls, n = 16). The rationale, organization, and recruitment of the subjects, biological procedures used have been described previously by Foli et al. [21]. In brief, 32 individuals were enrolled from April 2011 to March 2013 at the Yale-New Haven Hospital. Cases were matched for age, race, and gender to HIV-negative controls. At enrollment, the participant’s past medical history and demographic information were obtained. For the cases, we reviewed their medical records for medication history, HIV RNA copy number, and CD4 + T-cell count. The Human Apoptosis RT2 Profiler PCR Array kit (SuperArray Biosciences) was used to investigate apoptosis pathway-specific genes according to manufacturer’s instructions. The institutional review board of the Yale School of Medicine approved the study protocol.

### Statistical analysis

We previously analyzed the data and identified 26 out of 84 genes to be differentially expressed between the cases and controls [21]. We identified the 26 differentially expressed genes based on the false discovery rate (FDR) adjusted p-value using empirical Bayes moderated tests. In this secondary analysis, we sought to rank further the critical genes which contributed to profiling differences, using the maximal relevance and minimum redundancy algorithm. This algorithm chooses a subset of genes (features) having the most correlation with a class (relevance, the outcome) and the least correlation between themselves (redundancy), ranking features according to the minimal-redundancy-maximal-relevance criteria [22]. The F-statistic was used to calculate correlation with the class (relevance). For correlation between genes (redundancy), the Pearson correlation coefficient was used. Next, genes were selected one by one by applying a greedy search to maximize the objective function, a function that integrates relevance and redundancy information of each gene into a single scoring mechanism [22]. Once computed, the algorithm ranks the variables according to their importance score. We estimated the features or genes' predictive accuracy in distinguishing class membership (case vs. control) using several machine learning algorithms including Linear Discriminant Analysis (LDA), Quadratic Discriminant Analysis (QDA), K-Nearest Neighbor (KNN), Support Vector Machine (SVM), Classification Tree (CART), Adaboost (ADA), Neural Networks (NNET), Random Forest (RF), Gaussian process and Logistic Regression [23]. We used the leave-one-out cross-validation procedure to estimate the performance of the classifier algorithms. We used the algorithm with the highest cross-validated area under the receiver operating curves in evaluating the diagnostic performance of the mRMR genes as biomarkers of mitochondrial toxicity. Network analysis was used to estimate and visualize the relationship among the 26 genes. More specifically, we employed Markov Random Field (MRF) or undirected network models to identify network structures related to mitochondrial toxicity. The network analysis involved estimating network models, computing network centrality indices, evaluating the accuracy of the network structures, comparing the network structures of cases and controls, and using spin glass models to find communities in the network structures of cases and controls [24]. Statistical analyses were performed using the R package version 4.0.3 and SAS software version 9.4 (SAS Institute, Cary, North Carolina). All *P* values were 2-sided and considered statistically significant if < 0.05.

## Results

**Fig. 1:**
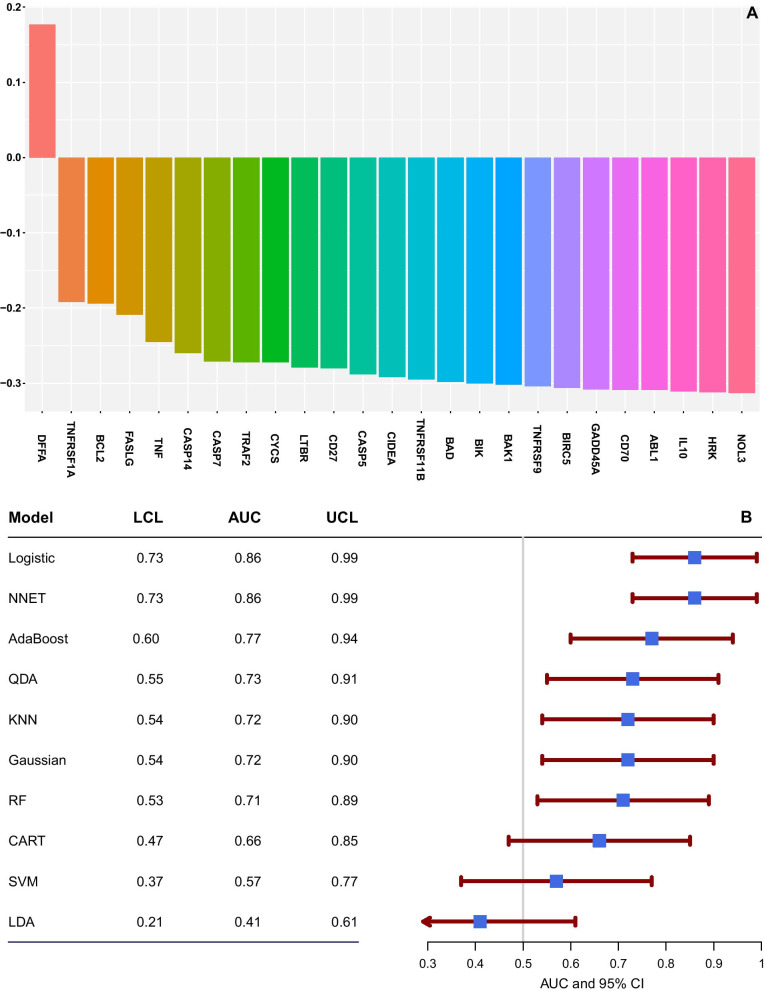
**A** Gene Importance Scores. DFFA (most relevant) and TNFRS1A (redundant) were the top two genes. The rest are shown in decreasing order of gene importance. **B** Cross-validated areas under the receiver operating curves together with their 95% confidence intervals. Ten classifier models for the top two mRMR genes. LDA: linear discriminant analysis, QDA: quadratic discriminant analysis, KNN: k-nearest neighbor, SVM: support vector machine, CART: classification tree, AdaBoost, NNET: neural networks, RF: random forest, Gaussian: Gaussian Process, Logit: logistic regression

The study included a total of 32 HIV-infected and HIV-uninfected participants. Seventy-eight percent of the participants were whites (n = 25), and the majority of them were males (n = 22, 69%) [21]. The median age of the study participants was 49.5 years (IQR = 33–66). In this study, we applied a maximum relevance minimum redundancy method to rank the importance of the 26 genes differentially expressed between the two groups. DFFA was the most relevant (positive score) and TNFRSF1A (redundant, least negative score), as shown in Fig. 1A. DFFA is a pro-apoptotic gene in the executioner pathway, and TNFRSF1A is a proapoptotic gene in the extrinsic pathway. To assess the discriminatory power of DFFA and TNFRSF1A, we then tested several classifier models to classify study participants based on these two selected genes into groups. Due to the smaller sample size, we used the leave-one-out cross-validation procedure to estimate the performance of the classifier models. We used the model with the highest cross-validated area under the receiver operating curve (ROC) in evaluating the diagnostic performance of the mRMR genes as biomarkers of mitochondrial toxicity. Figure 1B displays the cross-validated areas under the ROC together with their 95% confidence intervals. The classifier models resulted in cross-validated areas under the ROC curve ranging from 0.41 to 0.86. Logistic regression and neural network models had the highest performance. These two classifier models correctly classified 86% of the participants into their respective groups using the two-top ranked mRMR genes (Fig. 1B).

### Estimating networks

**Fig. 2:**
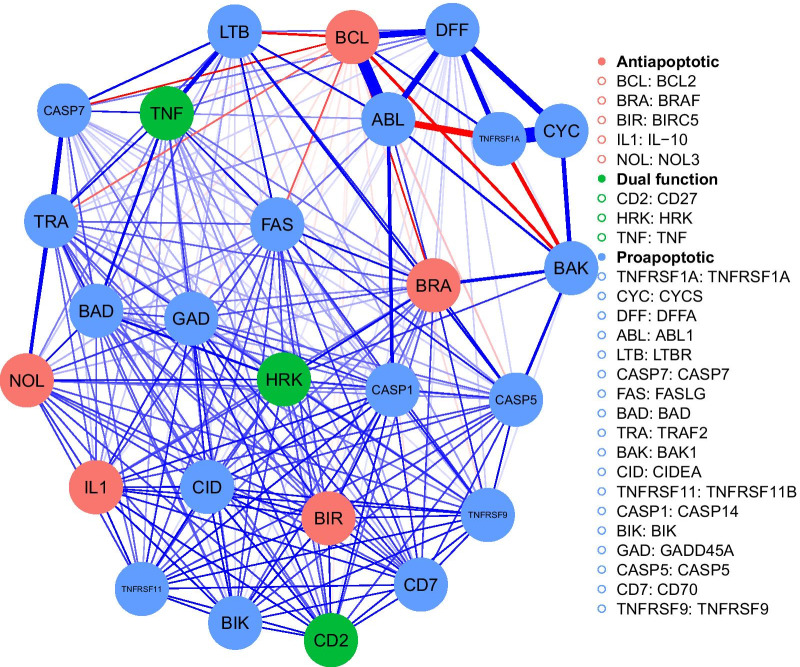
Network plot on the entire dataset. The circles represent nodes and the lines connecting them indicate edges, which are the partial correlation values between the nodes. Blue indicates positive and red indicates negative correlation value. The nodes were specified to show which were anti-apoptotic (red), pro-apoptotic (blue), and dual function (green)

We estimated a network of all the 26 genes, with edges connected by partial correlation values as shown in Fig. 2. We used a regularized Gaussian graphical model [25], which utilized graphical LASSO [26] in combination with tuning parameters selected by minimizing the Extended Bayesian Information Criterion [27] to estimate the networks. We generated network plot structures with nodes representing each of the individual genes. In the network plots, the width of the edges represented the strength of the connections; while, the blue or red edges illustrate positive or negative partial correlation values, respectively. Of the 26 genes, 18 were proapoptotic (TNFRSF1A, CYCS, DFFA, ABL1, LTBR, CASP7, FASLG, BAD, TRAF2, BAK1, CIDEA, TNFRSF11B, CASP14, BIK, GADD45A, CASP5, CD70, and TNFRSF9), 5 were antiapoptotic (BCL2, BRAF, BIRC5, IL-10, and NOL3), and 3 had dual functions (CD27, HRK, and TNF).

**Fig. 3:**
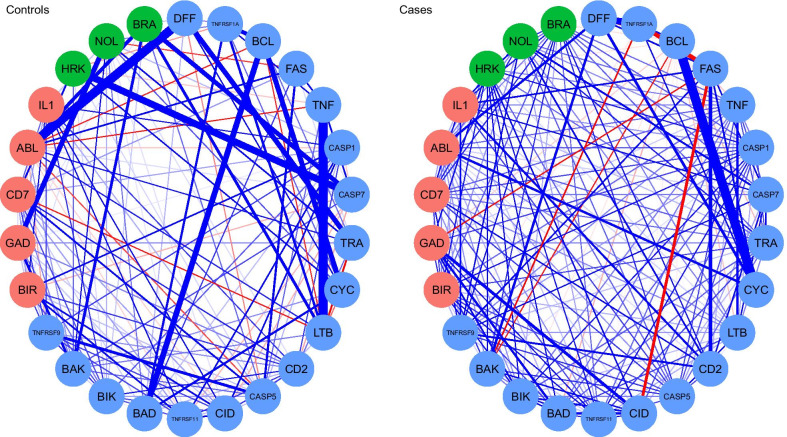
Network plots for cases and controls separately. Blue lines indicate positive partial correlation values, and red lines indicate negative partial correlation values. The thickness of the edges indicates the strength of the correlation

**Fig. 4:**
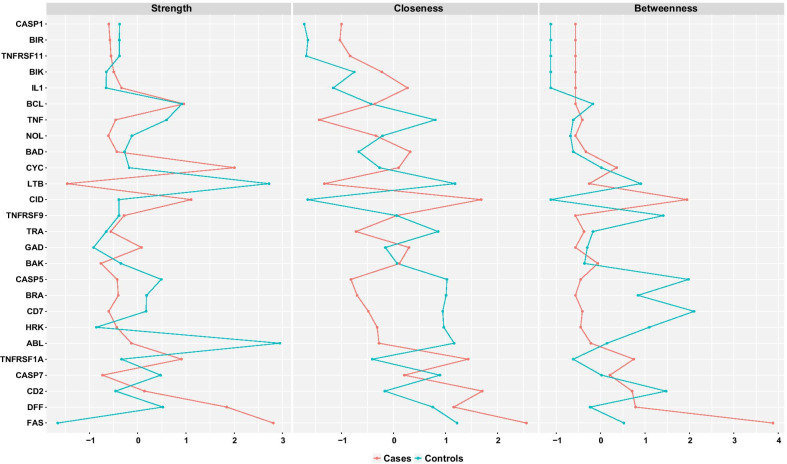
Centrality Plots. Cases are shown in red and controls in teal. FASLG had the highest strength, betweenness and closeness in cases while ABL had the highest strength, betweenness and closeness in controls

We also estimated the networks separately for cases and controls (Fig. 3). We obtained centrality measures and assessed the stability of networks for cases and controls. As shown in Fig. 4, we computed three different centrality indices: closeness, betweenness, and strength [28, 29]. Perturbations to the nodes with the highest closeness and betweenness may affect large parts of the network structure, and perturbations to the node with the highest strength might influence many other nodes and are therefore considered most important within the network structure. Further, we carried out accuracy tests to assess the stability of network structures for cases and controls. The accuracy tests included (a) estimation of the accuracy of the edge-weights by drawing bootstrapped confidence intervals (CIs); (b) investigating the stability of centrality indices after observing only portions of the data, and (c) performing bootstrapped difference tests between edge-weights and centrality indices to test whether these differ significantly from each other [24]. The case network structure (Fig. 3—right) showed the strongest positive edge-weights between DFFA and TNFRSF1A, CYCS and BCL2, and negative edge-weights between BCL2 and TNFRSF1A, BCL2 and FASLG, and FASLG and CIDEA. We evaluated the accuracy of connections by bootstrapped CIs analysis. The bootstrapped CIs for the estimated edge-weights were large, suggesting that many of the edge-weights did not differ significantly from one another. However, CIs for the edges of CD27 and FASLG, and FASLG and TNFRSF1A did not overlap with bootstrapped CIs of other edges and were likely the strongest edges. Centrality indices results revealed that FASLG had the highest strength, betweenness, and closeness (Fig. 4, red) among all 26 genes analyzed, suggesting that FASLG had most interactions with other genes in the network structure of the cases. In the controls network (Fig. 3, left), LTBR and TNF, DFFA and ABL1, HRK and CASP7, BCL2 and BAD, CYCS and BCL2 had strong positive edge-weights, with weak negative edge-weights found between TNF and ABL1, LTBR and CD70, CYCS and TRAF2, and CYCS and LTBR. As was the case with the network structure of the cases, the edge-weight accuracy results revealed that most of the edge-weights did not differ significantly from one another. Centrality indices plot (Fig. 4, teal) and centrality scores showed that ABL1 and LTBR were the most central genes in the controls network.

### Network comparison

To further analyze the overall differences between the two networks, a network comparison test (NCT) was performed to examine the differences in the weights of connection. The networks' overall connectivity (or global strength), defined as the weighted sum of the absolute connections, was calculated for cases and controls. To assess the difference in overall connectivity between networks of both groups, we implemented a permutation-based test for randomly regrouped genes [30, 31]. The test revealed that the overall connectivity was significantly different between the two networks (*p* < 0.05), and the controls network had more significant edge-weights between nodes compared to the cases.

### Community detection between cases and controls

**Fig. 5:**
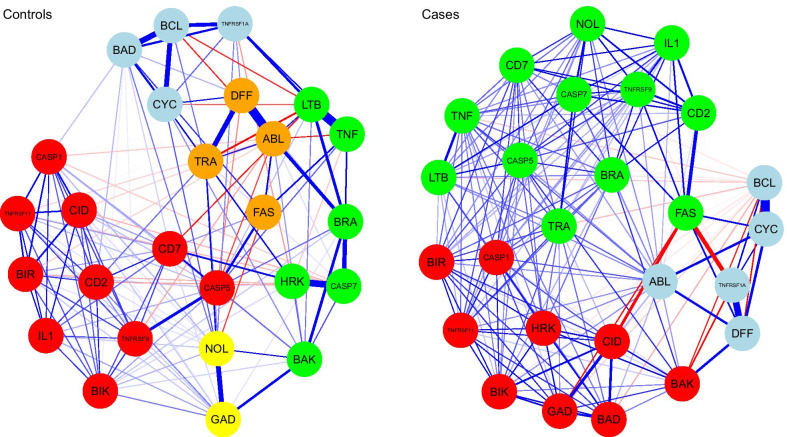
Spinglass algorithms showing clusters for cases and controls. There were 3 clusters for cases and 5 clusters in controls

We explored a network model-based clustering to find communities in the network graphs of cases and controls (Fig. 5). A community is a set of nodes with many edges inside the community and a few edges outside (i.e., between the community itself and the rest of the network graph) [32]. We used the Spinglass algorithm for community detection[32–34]. The algorithm identified 3 clusters in cases and 5 clusters in controls. For cases, DFFA, TNFRSF1A, BCL2, CYCS, and ABL1 were in cluster one (light blue in Fig. 5), FASLG, BRAF, NOL3, IL10, CD70, TNFRSF9, CASP5, CD27, LTBR, TRAF2, CASP7, and TNF belonged to cluster two (green in Fig. 5), and CASP14, HRK, GADD45A, BIRC5, BAK1, BIK, BAD, TNFRS11B, and CIDEA belonged to cluster three (light red in Fig. 5). For controls, DFFA, FASLG, TRAF, and ABL1 belonged to cluster one (orange in Fig. 5), CASP14, IL10, CD70, BIRC5, TNFRSF9, BIK, TNFRSF11B, CIDEA, CASP5, and CD27 were in cluster two (light red in Fig. 5), TNF, BRAF, HRK, BAK1, LTBR, and CASP7 were in cluster three (green in Fig. 5), TNFRSF1A, BAD, CYCS, and BCL2 were in cluster four (light blue in Fig. 5) and NOL3 and GADD45A were in cluster five (yellow in Fig. 5).

## Discussion

In this secondary analysis, we used 26 genes that we previously identified to be differentially expressed genes of the apoptosis pathway among HIV infected with toxicity and HIV uninfected participants. We applied a maximum relevance minimum redundancy algorithm on the 26 genes to rank critical genes contributing to profiling differences between cases and controls. The algorithm ranked DFFA and TNFRSF1A, two of the upregulated pro-apoptotic genes, on the top. We implemented several machine learning classifiers in evaluating the diagnostic performance of these two genes as biomarkers of mitochondrial toxicity. Due to the smaller sample size, leave-one-out cross-validation procedure was used to estimate the performance of the classifier models. We used the classifier model with the highest cross-validated area under the ROC curve. The cross-validated area under the ROC curve from the best model(s) was 0.86, thereby indicating the two genes correctly classified 86% of the study participants.

Since apoptosis is associated with almost all classes of ART, genes of the apoptotic pathway could serve as biomarkers for identifying and monitoring HIV treatment-experienced with ART-associated toxicity. Currently, there is no gold standard for diagnosing ART-induced mitochondrial toxicity. Diagnosis is based on a combination of clinical symptoms, laboratory testing, imaging studies, and, if available, a tissue biopsy to confirm mitochondrial damage. Confirmatory tissue biopsies are expensive, invasive, and not readily available. The use of the differentially expressed apoptotic genes could provide an accurate diagnosis of toxicity and eliminate the “trial and error” approach of switching around medications to relieve toxicity. Trial and error approach is expensive in the long run, as it favors the emergence of drug-resistant strains of HIV [35]. There is a need for a non-invasive, cost-effective biomarker for ART-induced mitochondrial toxicity to prevent unnecessary interruptions in ART and to guide the use of second-line regimens. If we could validate our findings in a larger cohort, a quantitative PCR assay of these apoptotic genes could serve as biomarkers for ART-induced toxicity.

Our network analysis findings of the proapoptotic FASLG gene being highly influential due to its high centrality in cases concur with several other physiologic studies that have found increased expression of FASLG in cases [35–37]. However, instead of FASLG, ABL1 and LTBR had the highest centrality in controls. We also observed that the number of more substantial edges (higher edge-weights) was lower in cases than the controls, suggesting that perturbations to the genes in cases network structure were incapable of affecting multiple nodes (genes), unlike in controls. Whether this suggests that HIV alters the protective dependent network structure of genes in controls requires further testing. We explored how genes were related to each other within the clusters of the Spinglass algorithms separately for cases and controls. For cases, the two most critical proapoptotic genes (DFFA, TNFRSF1A) selected by mRMR belonged to cluster 1, while within discrete clusters in the controls network structure. The fact that the two most important genes belonged to the same community (cluster 1) in cases along with a strong positive edge between two other proapoptic genes (CYCS and BCL2) warrants further exploration.

In homeostasis, genes of the apoptotic pathway (pro-and anti-apoptotic) work in tandem [38]. It is therefore interesting that we found a deferential clustering of pro- and anti-apoptotic genes between cases and control. Moreover, among the cases, we found less clustering compared to controls. Less clustering might suggest that cases with mitochondrial toxicity have perturbation of the apoptotic pathway favoring apoptosis. FASLG, ABL1, and LTBR, all proapoptotic genes, had the highest strength, betweenness, and closeness in cases or controls. FASLG is a member of a family of proteins that signals the initiation of a caspase cascade—a series of steps that result in apoptosis. This signaling is common to both extrinsic and intrinsic apoptotic pathways. Thus, the high degree of apoptosis in cases may be due to deploying of both extrinsic and intrinsic pathways. In response to oxidative stress, ABL1 and LTBR target the mitochondria and mediate mitochondrial dysfunction and apoptosis. The role of FASLG in apoptosis may be more global than the role of ABL1 or LTBR in apoptosis. The study has several limitations. First, as a cross-sectional study, we do not know the dynamic changes of these genes before and during ART. Second, we did not have a control group of HIV treatment-naive or HIV treatment-experienced individuals without toxicity. Third, the small sample size of study participants did not allow us to obtain patient-specific risks for the upregulation of these genes.

## Conclusions

This is a case–control study of the differentially expressed apoptosis pathway genes in HIV infected participants with mitochondrial toxicity and uninfected controls. We applied the maximum relevance minimum redundancy (mRMR) algorithm on the differentially expressed genes between the cases and controls. The mRMR algorithm ranked DFFA and TNFRSF1A, two of the upregulated proapoptotic genes, on the top. These two genes correctly classified 86% of the participants into their respective groups. Network analysis revealed that FASLG had the highest centrality in cases with ABL1 and LTBR in controls, with a new correlation of genes associated with mitochondrial toxicity. Our findings are consistent with other studies that suggest apoptosis may be a critical singnal in ART-induced mitochondrial toxicity. Future studies should validate the use of apoptotic genes, particularly DFFA and TNFRSF1A, as biomarkers of ART-associated toxicity.

## Data Availability

The datasets analyzed during the current study are available in the BMC Genomics Dataset repository, https://ragon.partners.org/musiebiostats/publications.html.

## Abbreviations

HIV: Human Immunodeficiency Virus
IQR: Interquartile range
mRMR: Maximum relevance minimum redundancy
ART: Antiretroviral therapy
PLWH: People living with HIV
NRTIs: Nucleoside reverse transcriptase inhibitors
mtDNA: Mitochondrial DNA
Pol-γ: Polymerase gamma
PIs: Protease inhibitors
NNRTIs: Non-nucleoside reverse transcriptase inhibitors
KNN: K-nearest neighbors
NCT: Network comparison test
LASSO: Least absolute shrinkage and selection operator
